# Microexcision of intramedullary schwannoma at the thoracic vertebra

**DOI:** 10.3892/etm.2013.890

**Published:** 2013-01-08

**Authors:** JIANWEN LI, YIQUAN KE, MIN HUANG, ZHIBIN LI, YI WU

**Affiliations:** 1 Department of Neurosurgery, Neurosurgery Institute, Key Laboratory on Brain Function Repair and Regeneration of Guangdong Province, Zhujiang Hospital of Southern Medical University, Guangzhou 510282;; 2 Neurosurgery Department of Jiangmen Central Hospital, Jiangmen, Guangdong 529030, P.R. China

**Keywords:** spinal cord, intramedullary schwannoma, treatment, follow-up

## Abstract

Intramedullary schwannoma is often misdiagnosed as other types of malignant tumour prior to surgery due to its atypical imaging appearance and low incidence. In the present study, a case of small intramedullary schwannoma was analysed using clinical and imaging data. Data concerning the surgery and follow-up process of this case were collected. Instead of performing the traditional surgical procedure of cutting the central and posterior rhizotomies of the patient, minimally invasive hemilaminectomy was performed to maintain spinal stability. This procedure was selected since the small mass would be removed completely via minimally invasive hemilaminectomy. Intramedullary schwannoma was confirmed following surgery. The patient recovered well and no recurrence of the tumour was detected during the two-year follow-up period. In conclusion, the treatment strategy for intramedullary schwannoma was determined based on its atypical symptoms and imaging characteristics.

## Introduction

Intramedullary schwannoma, which was first reported by Penfild in 1932 (1), is a rare tumour that accounts for 1.1% of all intraspinal tumours. Prior to surgery, intramedullary schwannoma is often misdiagnosed as other types of malignant tumour, including ependymoma, astrocytoma and hemangioblastoma, due to its atypical imaging appearance and low incidence. Long follow-ups of intramedullary schwannomas are unavailable.

The present study collected the surgery and follow-up process data of one case of intramedullary schwannoma.

## Case report

**Figure 1.**
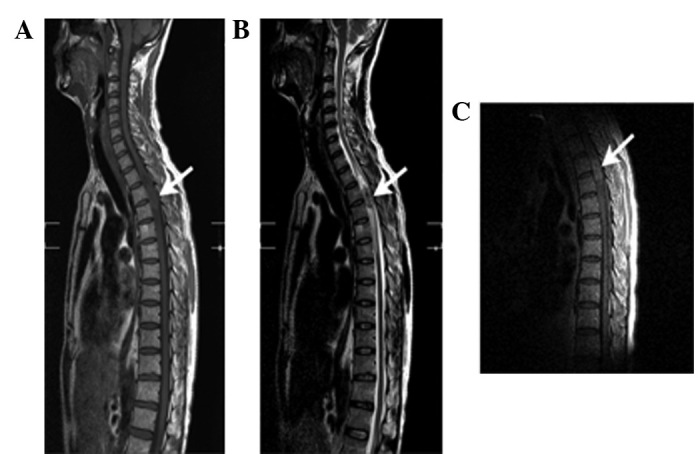
MRI images prior to surgery. (A) Abnormal ovoid long T1 and (B) long T2 soft tissue mass was observed at the level of third and fourth thoracic vertebra (white arrows). The size of the lesion was 1.3x1.1x2.4 cm with a clear margin and heterogenous intensity. (C) The lesion showed heterogenous contrast enhancement and compression of the adjacent subarachnoid space (white arrow).

A 42-year-old patient complaining of progressive zonesthesia in the right side of the chest, weakness and numbness of the bilateral lower limbs for 1.5 years and dysuria and paralysis for 1 week was admitted to Jiangmen Central Hospital. The present study was conducted in accordance with the Declaration of Helsinki and with the approval of the Ethics Committee of Zhujiang Hospital, Nanfang Medical University. Written informed consent was obtained from the patient. The physical examination revealed a disturbance of superficial sensation, grade 2 muscle strength and positive Babinski sign. Abnormal long ovoid T1 (Fig. 1A) and T2 (Fig. 1B) soft-tissue masses were observed at the third and fourth thoracic vertebrae. The lesion size was 1.3x1.1x2.4 cm with a clear margin and heterogenous intensity. The lesion showed heterogenous contrast enhancement and compression of the adjacent subarachnoid space (Fig. 1C). The spinal cord adjacent to the lesion was swollen. According to the MRI appearance of the lesion, astrocytoma or ependymoma was suspected. The possibility of a malignant tumour was also suspected due to a nodule observed on the left lung.

Tumour excision was initiated with exploration by decompression of the vertebral bone. After the patient’s relative signed the informed consent agreement, the surgery was performed as follows. Sterilised methylene blue fluid was used to create a marker for the X-ray at the third thoracic vertebra 1 day before the surgery. General anaesthesia was used and the patient was left in a recumbent position. A small incision was made to reveal the vertebral disc of the third and fourth thoracic vertebrae. The inferior third and superior fourth vertebral plates were drilled to open a 2x2-cm bone window. The whole surgery was performed under a microscope manufactured by Leica (Wetzlar, Germany). A tight adhesion of the spinal dura, arachnoid and spinal cord was observed after the spinal dura was cut. A fragile tumour supplied with rich blood was revealed in the spinal cord and the subarachnoid space. A malignant tumour was initially diagnosed, but the tumour was noted to have a clear margin with the spinal cord surrounded by proliferating small vessels. The lesion was excised completely.

Schwannoma was diagnosed by frozen section pathology. Following surgery, the numbness of the right side of the chest and weakness of the lower limbs were reduced compared with before the surgery.

**Figure 2.**
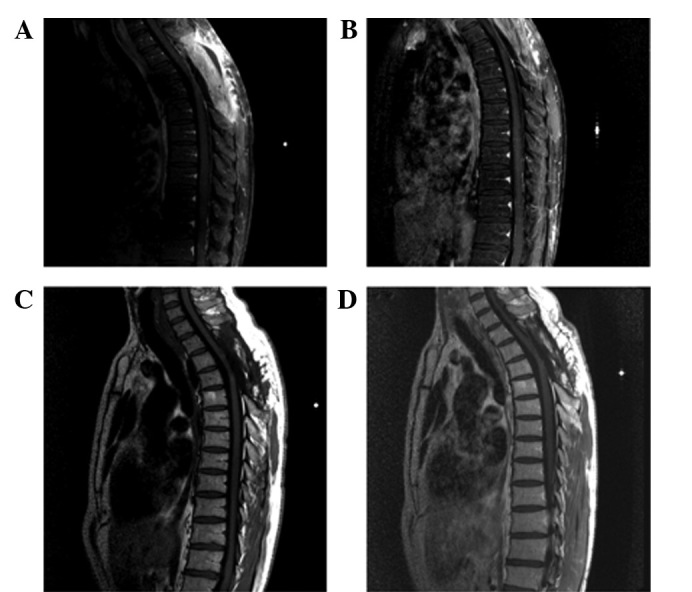
MRI images following tumour incision (T1-contrast). No lesion was observed in follow-up MRI at (A) 3 months, (B) 6 months, (C) 12 months and (D) 18 months after surgery. There was a small myelomalacia in the spinal cord.

No lesion was detected using MRI during the follow-up period at 3 (Fig. 2A), 6 (Fig. 2B), 12 (Fig. 2C) and 18 months (Fig. 2D) after the surgery. A small ariaosis was observed in the spinal cord.

The numbness of the right side of the chest had completely disappeared at 6 months after the surgery. The weakness and numbness of the lower limb partially improved. Although the patient continued to have defecation and micturition disturbances, all symptoms had disappeared at 18 months after the surgery. A small fluid-filled region was observed in the epidural space.

## Discussion

Intramedullary schwannoma is a rare tumour that accounts for ∼1.1% of schwannomas in the spinal canal. Up to 34 cases were included in the study of Hejazi and Hassler (2) published in 1998, whereas Qian *et al*(3) observed only 82 cases when reviewing domestic and foreign data in 2006. Ross *et al*(1) noted that 60.7 and 20% of all intramedullary schwannomas occurred in the cervical and thoracic regions, respectively (4). This type of tumour is uncommon in other regions. The ages of the patients in the reported cases ranged between 11 months and 53 year.

Based on the reported cases, the main symptoms of intramedullary schwannomas are progressive numbness, fatigue and pain in the extremities, whereas the main physical signs are hypertonia, decreased muscle strength, hypalgesia and hypopselaphesia, tenden reflex attenuation and tendinous reflex. These clinical manifestations are also common in other lesions of the spinal cord.

Intramedullary schwannoma has no specific imaging features (3). However, Kodama *et al*(5) reported that diagnosis of schwannoma should be considered if an intramedullary tumour has a clear boundary in the spinal cord and intense enhancement. Qian *et al*(3) described certain changes in MR that aid the diagnosis of intramedullary schwannoma. Firstly, the tumour exhibits isointensity or a slightly longer T1 and isointensity or long T2 signals, often combined with cystic degeneration. Secondly, the lesion shows intense and homogenous enhancement. Thirdly, the margin is clear. Fourthly, the lesions are often small (normally within three vertebrae) tumours. In 2005, Kim *et al*(6) revealed that slight peritumoural oedema is one of the characteristics of intramedullary schwannomas. However, another study did not agree with this finding (3).

Owing to its low incidence and lack of clinical and imaging manifestation, intramedullary schwannoma is often misdiagnosed as other types of intramedullary tumour such as ependymoma, astrocytoma, hemangioblastoma and subependymoma, among others (1,3,6).

No widely accepted explanation is available for the occurrence of intramedullary schwannoma. The origin of the tumour has multiple factors. Several possibilities based on the study by Liu *et al*(4) for the origin of the lesion are as follows: i) schwannoma cells in the posterior spinal cord nerve root; ii) schwannoma cells located along the blood vessels and peripheral nerves of the spinal cord; iii) schwannoma cells dislocated during the closure of the neural crest in the fourth week of embryonic development; iv) pia mater cells from the mesoderm; v) the peripheral fibres of vagus nerves in the spinal cord; and vi) a traumatic spinal cord injury or a chronic disorder of the central nervous system (1,7,8).

The traditional surgical approach for removing occupying lesions in the spinal canal causes adverse effects in three column structures. The spinous process, supraspinal and interspinous ligaments, vertebral arch, vertebral plate, *ligamentum flavum* and facet joint are cut during central and posterior rhizotomies, resulting in spinal instability and thus significantly affecting the lives of patients.

According to Denis’s three-column principle (9), minimally invasive hemilaminectomy should be used as the treatment method for space-occupying lesions instead of central and posterior rhizotomies to maintain the stability of the vertebral column (10). In the presence of clear peripheral oedema, malignant tumours diagnosed prior to surgery should be treated using central and posterior rhizotomies. In addition, a biopsy should be performed to confirm the diagnosis or to partly excise the tumour. These procedures affect the stability of the vertebral column.

This case was preoperatively diagnosed as an ependymoma or astrocytoma. Minimally invasive hemilaminectomy was performed on the space-occupying lesion in the spinal cord with clear boundaries and intense enhancement. The possibility of endoscopic resection was also considered. The surgery was considered to be successful. All the benign tumours were removed and central and posterior rhizotomies were avoided. The patient recovered well after the surgery and intramedullary schwannoma did not recur during the two-year follow-up period.

Therefore, depending on the patient’s condition, doctors may consider a case to be a possible intramedullary schwannoma and perform minimally invasive hemilaminectomy if no confirmed diagnosis has been acheived prior to the surgery, particularly when differential diagnosis exists among ependymoma, astrocytoma and hemangioblastoma and if the space-occupying lesion in the spinal cord had clear boundaries and exhibited intense enhancement. The necessity of cutting the spinous process, ligamentum flavum and bi-vertebral plate may then be decided after the biopsy results are known. In this way, the spinal instability resulting from the traditional surgical procedure of excision during central and posterior rhizotomies, which are preoperatively applied to patients diagnosed with malignant tumours, may be avoided.
